# Supplementary material for: Prefrontal Cortex Hemodynamics and Functional Connectivity Changes during Performance Working Memory Tasks in Older Adults with Sleep Disorders
Source: Brain Sci. 2023 Mar 15;13(3):497. doi: 10.3390/brainsci13030497 (PMC10046575; doi:10.3390/brainsci13030497)
Supplement: Supplementary file 1 [file brainsci-13-00497-s001.zip › brainsci-2234344-supplementary.pdf]

brain sciences

MDPI

Supplementary

Prefrontal Cortex Hemodynamics and Functional Connectivity Changes During Performance Working Memory Tasks in Older Adults with Sleep Disorders

Jiahui Gao[1†], Lin Zhang[1†], Jingfang Zhu[1], Zhenxing Guo[1], Miaoran Lin[1], Linxin Bai[1], Peiyun Zheng[1], Weilin Liu[1], Jia Huang[1] and Zhizhen Liu[1,2*]

[1]College of Rehabilitation Medicine, Fujian University of Traditional Chinese Medicine, Fuzhou, Fujian 350122, China;
[2]National-Local Joint Engineering Research Center of Rehabilitation Medicine Technology, Fujian University of Traditional Chinese Medicine, Fuzhou, Fujian 350122, China

HbR were significantly different in channel 3 (located in DLPFC) and channel 40(located in FPC) during the 2-back task, and both showed higher levels in the SD group. HbT was significantly different in channel 8(located in DLPFC) during 0-back 、1-back and 2-back tasks, and both showed smaller values in the SD group. While HbO is more sensitive and dependable than HbR， so only the results of HbR were discussed in this study.

Scheme 1. Oxygenated hemoglobin concentration changes in the HC and SD performing 0-back.

Brain regions	Channel	SD group	HC group	t/Z	*P*-value	FDR	Effect Size
DLPFC	CH1	-0.13±0.27	-0.05±0.26	-1.00	0.32	0.66	−0.24
DLPFC	CH2	-0.06±0.26	0.04±0.32	-1.96	0.05	0.66	−0.46
DLPFC	CH3	-0.18(-0.32±0.04)	-0.12(-0.31±0.03)	0.27	0.80	0.93	-0.04
DLPFC	CH4	-0.13±0.33	-0.03±0.28	-1.78	0.08	0.66	−0.41
DLPFC	CH5	0.08(-0.26±0.36)	-0.09(-0.29±0.21)	1.41	0.16	0.66	0.20
DLPFC	CH7	0.05±0.77	0.01±0.54	0.14	0.89	0.93	0.03
DLPFC	CH8	-0.15±0.36	0.00±0.35	-1.15	0.26	0.66	−0.27
DLPFC	CH9	-0.15±0.23	-0.01±0.34	-2.3	0.02	0.66	−0.54
DLPFC	CH10	-0.13(-0.42±0.15)	-0.06(-0.32±0.28)	1.40	0.16	0.66	-0.19
DLPFC	CH11	-0.04±0.28	0.01±0.26	-0.99	0.32	0.66	−0.23

DLPFC	CH12	-0.03(-0.22±0.11)	0.02(-0.27±0.12)	0.97	0.33	0.66	-0.13
DLPFC	CH13	-0.14(-0.32±0.16)	-0.08(-0.26±0.11)	0.42	0.68	0.93	-0.06
DLPFC	CH14	-0.16±0.33	-0.09±0.27	-0.99	0.33	0.66	−0.23
DLPFC	CH19	-0.01(-0.34-0.23)	-0.05(-0.15-0.06)	-1.09	0.28	0.66	0.15
DLPFC	CH23	0.22±0.84	0.04±0.55	1.21	0.23	0.66	0.28
DLPFC	CH29	-0.03(-0.31±0.24)	0.04(-0.31±0.29)	0.98	0.33	0.66	-0.13
DLPFC	CH34	-0.07±0.45	-0.13±0.35	0.62	0.54	0.91	0.15
DLPFC	CH50	-0.26(-0.56±0.20)	-0.31(-0.69±0.04)	-0.50	0.62	0.93	0.07
DLPFC	CH55	0.29±0.87	0.10±0.67	0.98	0.33	0.66	0.23
VLPFC	CH17	-0.14(-0.38±0.19)	-0.10(-0.32±0.13)	0.40	0.69	0.93	-0.05
VLPFC	CH18	-0.01(-0.22±0.32)	-0.03(-0.35±0.16)	1.32	0.19	0.66	0.18
VLPFC	CH24	0.00(-0.43±0.24)	0.14(-0.29±0.52)	1.39	0.17	0.66	-0.19
VLPFC	CH25	0.29(-0.38±0.88)	0.17(-0.11±0.80)	-0.37	0.71	0.93	0.05
VLPFC	CH28	-0.04±0.30	-0.07±0.30	0.14	0.89	0.93	0.03
VLPFC	CH35	-0.15±0.65	-0.12±0.49	-0.15	0.88	0.93	−0.03
VLPFC	CH39	0.29±1.05	0.18±0.66	0.41	0.69	0.93	0.10
VLPFC	CH45	0.15(-0.20±0.39)	-0.08(-0.42±0.40)	-1.02	0.31	0.66	0.14
FPC	CH21	0.09±0.63	-0.03±0.55	1.19	0.24	0.66	0.28
FPC	CH22	0.20±0.87	0.11±0.68	0.54	0.59	0.93	0.13
FPC	CH30	-0.07±0.37	-0.03±0.37	-0.09	0.93	0.95	−0.02
FPC	CH31	-0.04±0.45	-0.08±0.45	-0.16	0.87	0.93	−0.04
FPC	CH32	-0.09±0.3	-0.07±0.29	-0.15	0.88	0.93	−0.04
FPC	CH33	-0.11±0.37	-0.09±0.33	0.02	0.98	0.98	0.01
FPC	CH40	0.07(-0.28±0.40)	0.14(-0.12±1.52)	1.64	0.10	0.66	-0.22

FPC	CH41	0.16(-0.09±0.46)	-0.02(-0.39±0.62)	-0.84	0.40	0.73	0.11
FPC	CH42	0.15±0.77	0.13±0.54	0.23	0.82	0.93	0.05
FPC	CH43	0.19(-0.27±0.63)	0.04(-0.38±0.55)	-0.78	0.43	0.75	0.11
FPC	CH44	0.04(-0.41±0.47)	-0.04(-0.49±0.48)	-0.43	0.67	0.93	0.06
OFC	CH51	-0.29(-0.57±0.35)	-0.20(-0.63±0.11)	0.31	0.76	0.93	0.04
OFC	CH52	-0.07±0.52	-0.24±0.50	1.82	0.07	0.66	0.42
OFC	CH53	-0.14±0.54	-0.2±0.43	1.03	0.31	0.66	0.24
OFC	CH54	0.09±0.58	0.01±0.48	0.85	0.4	0.73	0.20

Scheme 2. Oxygenated hemoglobin concentration changes in the HC and SD performing 1-back.

Brain regions	Channel	SD group	HC group	t/Z	*P*-value	FDR	Effect Size
DLPFC	CH1	-0.12±0.32	-0.01±0.28	-1.55	0.13	0.55	−0.36
DLPFC	CH2	-0.06±0.37	0.07±0.31	-1.83	0.07	0.55	−0.43
DLPFC	CH3	-0.06(-0.40±0.17)	-0.10(-0.35±0.12)	-0.25	0.80	0.88	0.03
DLPFC	CH4	-0.10±0.42	-0.01±0.26	-1.14	0.26	0.67	−0.26
DLPFC	CH5	-0.00(-0.34-0.34)	-0.07(-0.34-0.35)	-0.18	0.86	0.90	0.02
DLPFC	CH7	0.21(-0.31-0.89)	0.07(-0.34-0.57)	-0.61	0.54	0.83	0.08
DLPFC	CH8	0.07(-0.21±0.26)	0.17(-0.06±0.43)	2.06	0.04	0.55	-0.28
DLPFC	CH9	-0.11(-0.37±0.16)	0.02(-0.14±0.19)	1.56	0.12	0.55	-0.21
DLPFC	CH10	0.06±0.38	0.1±0.49	-0.23	0.82	0.88	−0.05
DLPFC	CH11	-0.04(-0.23±0.15)	0.03(-0.24±0.16)	0.24	0.81	0.88	-0.03
DLPFC	CH12	-0.02±0.36	-0.05±0.25	-0.11	0.91	0.93	−0.03
DLPFC	CH13	-0.04(-0.30±0.30)	-0.15(-0.30±0.04)	-1.48	0.14	0.55	0.20
DLPFC	CH14	0.01±0.42	-0.07±0.32	0.42	0.67	0.87	0.10

DLPFC	CH19	0.09±0.54	-0.06±0.43	1.57	0.12	0.55	0.37
DLPFC	CH23	0.28(-0.03-0.67)	0.22(-0.20-0.60)	-0.57	0.57	0.85	0.08
DLPFC	CH29	0.15(-0.18±0.40)	0.03(-0.22±0.30)	-1.02	0.31	0.67	0.14
DLPFC	CH34	0.15(-0.34±0.30)	-0.05(-0.27±0.23)	-0.50	0.62	0.86	0.07
DLPFC	CH50	0.01(-0.36±0.36)	-0.16(-0.48±0.22)	-0.98	0.33	0.67	0.13
DLPFC	CH55	0.46±0.81	0.32±0.88	0.32	0.75	0.88	0.08
VLPFC	CH17	0.04(-0.37±0.44)	0.10(-0.14±0.25)	0.27	0.79	0.88	-0.04
VLPFC	CH18	0.15(-0.32±0.44)	-0.02(-0.31±0.37)	0.51	0.61	0.86	0.07
VLPFC	CH24	0.22(-0.22-0.62)	0.23(-0.10-0.80)	0.80	0.43	0.71	−0.11
VLPFC	CH25	0.65(0.28±0.99)	0.54(-0.11±0.97)	-1.00	0.32	0.67	0.14
VLPFC	CH28	0.07±0.38	-0.05±0.35	1.07	0.29	0.67	0.25
VLPFC	CH35	0.20(-0.33±0.55)	0.17(-0.17±0.46)	-0.45	0.65	0.87	0.06
VLPFC	CH39	0.58(0.16-1.49)	0.27(-0.09-1.07)	-1.36	0.18	0.65	0.18
VLPFC	CH45	0.5±0.63	0.27±0.88	1.05	0.3	0.67	0.25
FPC	CH20	0.36(0.12±0.82)	0.07(-0.38±0.47)	-1.93	0.05	0.55	0.26
FPC	CH21	0.07(-0.28±0.37)	0.05(-0.37±0.56)	-0.09	0.93	0.93	0.01
FPC	CH22	0.35(-0.03±0.71)	0.20(-0.28±0.69)	-0.92	0.36	0.67	0.13
FPC	CH30	0.10±0.47	-0.01±0.46	0.86	0.40	0.71	0.20
FPC	CH31	0.20(-0.06±0.45)	0.12(-0.25±0.38)	-1.18	0.24	0.67	0.16
FPC	CH32	0.04±0.44	0.00±0.27	0.30	0.76	0.88	0.07
FPC	CH33	0.09±0.49	-0.02±0.40	0.82	0.42	0.71	0.19
FPC	CH40	0.28(-0.20-0.57)	0.40(-0.18-0.76)	0.91	0.36	0.67	−0.12
FPC	CH41	0.35(-0.23±0.83)	0.15(-0.22±0.69)	-1.03	0.30	0.67	0.14
FPC	CH42	0.07±0.38	0.32±0.81	0.28	0.78	0.88	0.06

FPC	CH43	0.43(-0.13-0.74)	0.00(-0.22-0.74)	-1.28	0.20	0.66	0.17
FPC	CH44	0.28±0.99	0.11±0.99	0.63	0.53	0.83	0.15
OFC	CH51	0.07(-0.28±0.52)	-0.14(-0.48±0.20)	-1.58	0.11	0.55	0.21
OFC	CH52	0.24(-0.13±0.60)	0.09(-0.50±0.35)	-1.71	0.09	0.55	0.23
OFC	CH53	0.15(-0.12±0.45)	-0.05(-0.30±0.27)	-1.77	0.08	0.55	0.24
OFC	CH54	0.47(0.21±1.09)	0.16(-0.16±0.85)	-1.57	0.12	0.55	0.21

Scheme 3. Oxygenated hemoglobin concentration changes in the HC and SD performing 2-back.

Brain regions	Channel	SD group	HC group	t/Z	*P*-value	FDR	Effect Size
DLPFC	CH1	-0.18±0.42	-0.02±0.26	-1.77	0.08	0.57	−0.41
DLPFC	CH2	-0.09(-0.37±0.14)	-0.03(-0.17±0.38)	1.98	0.05	0.54	-0.27
DLPFC	CH3	-0.13±0.43	-0.04±0.32	-0.13	0.90	0.93	−0.03
DLPFC	CH4	-0.19±0.38	0.02±0.32	-2.48	0.02	0.43	−0.58
DLPFC	CH5	0.07(-0.08±0.37)	o.07(-0.45±0.35)	-1.01	0.31	0.74	0.14
DLPFC	CH7	0.30(0.06-0.88)	0.29(-0.17-0.86)	-0.36	0.72	0.93	0.05
DLPFC	CH8	-0.15±0.35	0.18±0.39	-3.94	**＜0.01***	**＜0.01***	−0.92
DLPFC	CH9	-0.18±0.40	0.01±0.43	-2.02	0.05	0.54	−0.47
DLPFC	CH10	-0.09±0.47	0.09±0.57	-1.45	0.15	0.69	−0.34
DLPFC	CH11	-0.08(-0.29±0.08)	0.00(-0.18±0.25)	1.58	0.11	0.68	-0.21
DLPFC	CH12	-0.08(-0.27±0.12)	-0.04(-0.23±0.15)	0.72	0.47	0.78	-0.10
DLPFC	CH13	-0.16(-0.49±0.23)	-0.13(-0.38±0.09)	0.65	0.52	0.83	-0.09
DLPFC	CH14	-0.04(-0.34±0.09)	-0.06(-0.27±0.20)	0.78	0.43	0.78	-0.11
DLPFC	CH19	0.03(-0.44-0.26)	0.02(-0.16-0.34)	0.27	0.79	0.93	−0.04
DLPFC	CH23	0.50(0.01±0.95)	0.41(-0.06±1.15)	-0.48	0.63	0.92	0.07

DLPFC	CH29	0.05±0.48	0.11±0.61	-0.73	0.47	0.78	−0.17
DLPFC	CH34	-0.01±0.41	0.05±0.46	-0.74	0.46	0.78	−0.17
DLPFC	CH50	-0.04±0.65	-0.13±0.68	0.11	0.91	0.93	0.03
DLPFC	CH55	0.94±1.08	0.58±1.16	1.25	0.22	0.74	0.29
VLPFC	CH17	-0.01(-0.36-0.26)	0.07(-0.12-0.23)	1.06	0.29	0.74	−0.14
VLPFC	CH18	0.08(-0.23±0.31)	0.11(-0.24±0.43)	0.29	0.78	0.93	-0.04
VLPFC	CH24	0.43±0.97	0.64±0.68	-1.54	0.13	0.69	−0.36
VLPFC	CH25	1.07(0.43±1.66)	0.73(0.31±1.40)	-1.10	0.27	0.74	0.15
VLPFC	CH28	0.01±0.41	-0.07±0.44	0.31	0.76	0.93	0.07
VLPFC	CH35	0.14±0.58	0.14±0.72	-0.17	0.87	0.93	−0.04
VLPFC	CH39	1.29±1.12	1.00±1.1	0.93	0.36	0.74	0.22
VLPFC	CH45	0.51(0.13±1.20)	0.28(-0.21±0.74)	-1.80	0.07	0.57	0.24
FPC	CH20	0.46(-0.05±0.68)	0.31(-0.12±0.93)	-0.02	0.99	0.99	0.00
FPC	CH21	0.22(-0.04-0.48)	0.11(-0.42-0.48)	-0.95	0.34	0.74	0.13
FPC	CH22	0.73±0.88	0.56±0.87	0.93	0.35	0.74	0.22
FPC	CH30	0.03±0.42	0.03±0.43	-0.32	0.75	0.93	−0.07
FPC	CH31	0.16±0.54	0.04±0.63	0.44	0.66	0.92	0.10
FPC	CH32	0.00±0.35	-0.01±0.39	-0.19	0.85	0.93	−0.05
FPC	CH33	-0.05±0.41	0.03±0.47	-1.03	0.31	0.74	−0.24
FPC	CH40	0.37±0.63	0.63±0.98	-1.33	0.19	0.74	−0.31
FPC	CH41	0.66±1.04	0.47±0.85	0.89	0.38	0.74	0.21
FPC	CH42	0.67±0.89	0.56±0.78	0.46	0.65	0.92	0.11
FPC	CH43	0.61(0.09±1.23)	0.32(-0.21±1.20)	-1.40	0.16	0.69	0.19
FPC	CH44	0.56±1.11	0.27±1.08	1.10	0.28	0.74	0.26

OFC	CH51	0.10(-0.47±0.35)	-0.02(-0.48±0.40)	-0.12	0.90	0.93	0.02
OFC	CH52	0.18(-0.03±0.40)	0.18(-0.03±0.40)	-0.92	0.36	0.74	0.13
OFC	CH53	0.13(-0.13±0.46)	0.01(-0.29±0.40)	-0.45	0.65	0.92	0.06
OFC	CH54	0.76±0.8	0.7±0.83	0.25	0.81	0.93	0.06

Scheme 4. Deoxygenated hemoglobin concentration changes in the HC and SD performing 0-back.

Brain regions	Channel	SD group	HC group	t/Z	*P*-value	FDR	Effect Size
DLPFC	CH1	0.04(-0.05±0.11)	0.02(-0.04±0.10)	-0.34	0.73	0.91	0.05
DLPFC	CH2	0.05(-0.06±0.15)	0.03(-0.07±0.28)	0.53	0.60	0.91	-0.07
DLPFC	CH3	-0.01(-0.05±0.17)	-0.02(-0.12±0.08)	-1.49	0.14	0.76	0.20
DLPFC	CH4	0.05±0.14	0.03±0.10	-0.51	0.61	0.91	−0.12
DLPFC	CH5	0.03(-0.14±0.22)	-0.02(-0.25±0.16)	-0.83	0.41	0.84	0.11
DLPFC	CH7	-0.16(-0.46±0.29)	-0.14(-0.34±0.08)	0.36	0.72	0.91	-0.05
DLPFC	CH8	-0.07(-0.16±0.12)	0.04(-0.12±0.15)	1.27	0.20	0.76	-0.17
DLPFC	CH9	-0.01(-0.11±0.12)	0.01(-0.07±0.13)	0.41	0.69	0.91	-0.06
DLPFC	CH10	-0.06±0.22	-0.01±0.20	-1.08	0.28	0.80	−0.25
DLPFC	CH11	0.04±0.16	0.07±0.11	-1.28	0.2	0.76	−0.30
DLPFC	CH12	0.03±0.14	0.03±0.09	-0.93	0.36	0.84	−0.22
DLPFC	CH13	0.01(-0.09-0.12)	0.01(-0.04-0.10)	0.12	0.90	0.96	−0.02
DLPFC	CH14	0.02(-0.08±0.13)	-0.03(-0.19±0.09)	-2.18	0.03	0.76	0.29
DLPFC	CH19	0.08±0.35	-0.01±0.24	1.09	0.28	0.80	0.25
DLPFC	CH23	0.09(-0.18±0.32)	0.05(-0.10±0.19)	-0.32	0.75	0.91	0.04
DLPFC	CH29	-0.12(-0.18±0.05)	0.00(-0.08±0.08)	2.11	0.04	0.76	-0.29
DLPFC	CH34	-0.03±0.15	0.02±0.15	-1.30	0.20	0.76	−0.30

DLPFC	CH50	-0.04±0.27	-0.05±0.16	0.50	0.62	0.91	0.12
DLPFC	CH55	-0.01(-0.26±0.19)	0.07(-0.12±0.26)	1.23	0.22	0.76	-0.17
VLPFC	CH17	0±0.16	0.01±0.15	-0.42	0.68	0.91	−0.10
VLPFC	CH18	0.00(-0.13±0.13)	-0.06(-0.18±0.05)	-1.37	0.17	0.76	0.19
VLPFC	CH24	-0.03(-0.43-0.15)	-0.06(-0.19-0.10)	0.22	0.83	0.92	−0.03
VLPFC	CH25	0.08(-0.18-0.42)	-0.03(-0.15-0.26)	-0.25	0.80	0.92	0.03
VLPFC	CH28	-0.05(-0.10±0.14)	-0.03(-0.12±0.07)	-0.03	0.98	0.98	0.00
VLPFC	CH35	0.06(-0.11-0.16)	0.03(-0.02-0.15)	0.08	0.94	0.96	−0.01
VLPFC	CH39	0.00(-0.26±0.20)	0.06(-0.10±0.32)	1.26	0.21	0.76	-0.17
VLPFC	CH45	-0.04±0.40	0.06±0.33	-1.39	0.17	0.76	−0.32
FPC	CH20	0.05(-0.39±0.20)	-0.06(-0.25±0.18)	0.08	0.94	0.96	-0.01
FPC	CH21	0.03±0.41	-0.05±0.25	1.23	0.22	0.76	0.29
FPC	CH22	0.01±0.33	-0.05±0.30	1.40	0.17	0.76	0.325
FPC	CH30	0.03±0.17	0.04±0.14	0.56	0.57	0.91	0.13
FPC	CH31	-0.02±0.19	0.02±0.16	-0.89	0.38	0.84	−0.21
FPC	CH32	0.04±0.15	0.02±0.14	-0.31	0.76	0.91	−0.07
FPC	CH33	0.01±0.17	0.03±0.13	-0.85	0.4	0.84	−0.20
FPC	CH40	-0.11(-0.33-0.12)	-0.15(-2.13-0.07)	-1.21	0.23	0.76	0.16
FPC	CH41	-0.04(-0.42±0.20)	-0.05(-0.19±0.16)	0.37	0.71	0.91	-0.05
FPC	CH42	-0.02(-0.30±0.21)	0.11(-0.26±0.16)	-0.70	0.49	0.88	0.09
FPC	CH43	-0.05(-0.27-0.20)	0.03(-0.12-0.38)	0.98	0.33	0.84	−0.13
FPC	CH44	-0.06(-0.27±0.31)	0.01(-0.19±0.19)	0.73	0.47	0.88	-0.10
OFC	CH51	-0.01(-0.14±0.18)	-0.01(-0.12±0.08)	-0.24	0.81	0.92	0.03
OFC	CH52	-0.03(-0.19±0.15)	-0.02(-0.38±0.09)	-0.74	0.46	0.88	0.10

OFC	CH53	-0.11±0.19	-0.08±0.17	0.31	0.76	0.91	0.07
OFC	CH54	-0.05(-0.22±0.10)	0.00(-0.16±0.14)	0.86	0.39	0.84	-0.12

Scheme 5. Deoxygenated hemoglobin concentration changes in the HC and SD performing 1-back.

Brain regions	Channel	SD group	HC group	t/Z	*P*-value	FDR	Effect Size
DLPFC	CH1	-0.03(-0.12±0.13)	-0.02(-0.10±0.03)	-0.84	0.40	0.91	0.11
DLPFC	CH2	0.03(-0.12±0.13)	0.01(-0.07±0.08)	0.60	0.55	0.91	-0.08
DLPFC	CH3	-0.01(-0.08-0.15)	-0.03(-0.11-0.08)	-0.65	0.51	0.91	0.09
DLPFC	CH4	-0.02(-0.10±0.08)	-0.01(-0.05±0.08)	0.65	0.51	0.91	-0.09
DLPFC	CH5	-0.03(-0.21±0.15)	-0.07(-0.23±0.06)	-1.13	0.26	0.91	0.15
DLPFC	CH7	-0.08(-0.30±0.27)	-0.13(-0.29±0.09)	-0.74	0.46	0.91	0.10
DLPFC	CH8	-0.04(-0.21-0.06)	0.02(-0.14-0.12)	1.38	0.17	0.91	−0.20
DLPFC	CH9	-0.06(-0.16-0.03)	-0.03(-0.13-0.05)	0.90	0.37	0.91	−0.12
DLPFC	CH10	-0.07±0.20	-0.05±0.19	-0.47	0.64	0.93	−0.11
DLPFC	CH11	-0.04(-0.14±0.02)	0.02(-0.06±0.11)	2.37	0.02	0.43	-0.32
DLPFC	CH12	-0.03(-0.11±0.12)	0.00(-0.08±0.06)	0.67	0.51	0.91	-0.09
DLPFC	CH13	-0.03(-0.12±0.05)	0.03(-0.09±0.10)	0.86	0.39	0.91	-0.12
DLPFC	CH14	0.00±0.22	-0.04±0.17	0.93	0.36	0.91	0.22
DLPFC	CH19	-0.02(-0.10-0.27)	-0.06(-0.19-0.05)	-1.38	0.17	0.91	0.19
DLPFC	CH23	0.03(-0.19±0.25)	0.02(-0.21±0.19)	0.31	0.76	0.96	0.04
DLPFC	CH29	-0.09(-0.25±0.00)	-0.07(-0.17±0.03)	1.22	0.22	0.91	0.16
DLPFC	CH34	-0.05(-0.20-0.04)	0.01(-0.16-0.09)	0.82	0.41	0.91	−0.11
DLPFC	CH50	-0.11(-0.29±0.02)	-0.12(-0.24±0.05)	0.15	0.88	0.97	-0.02
DLPFC	CH55	0.05±0.46	0.07±0.46	0.26	0.80	0.96	0.06

VLPFC	CH17	-0.08(-0.25±0.16)	-0.08(-0.17±0.05)	-0.20	0.84	0.97	0.03
VLPFC	CH18	-0.01(-0.22±0.18)	-0.08(-0.20±0.03)	-1.31	0.19	0.91	0.18
VLPFC	CH24	-0.22±0.61	-0.10±0.42	-0.88	0.38	0.91	−0.21
VLPFC	CH25	-0.10±0.50	-0.05±0.63	0.07	0.94	0.98	0.02
VLPFC	CH28	-0.01±0.17	-0.06±0.15	0.61	0.55	0.91	0.14
VLPFC	CH35	-0.02(-0.12-0.12)	0.01(-0.11-0.18)	0.46	0.65	0.93	−0.06
VLPFC	CH39	0.04(-0.26±0.45)	0.11(-0.04±0.32)	0.89	0.37	0.91	-0.12
VLPFC	CH45	0.14(-0.09±0.37)	0.12(-0.11±0.26)	-0.50	0.62	0.93	0.07
FPC	CH20	-0.01(-0.27-0.31)	0.02(-0.28-0.26)	0.03	0.98	0.98	−0.00
FPC	CH21	-0.04±0.31	-0.12±0.29	1.15	0.25	0.91	0.27
FPC	CH22	-0.02(-0.19±0.23)	-0.09(-0.37±0.13)	-1.57	0.12	0.91	0.21
FPC	CH30	-0.03±0.2	-0.03±0.25	0.03	0.98	0.98	0.01
FPC	CH31	-0.03(-0.15±0.08)	0.00(-0.16±0.13)	0.72	0.47	0.91	-0.10
FPC	CH32	0.00±0.22	-0.01±0.14	-0.39	0.70	0.96	−0.09
FPC	CH33	-0.03(-0.11±0.09)	-0.03(-0.12±0.10)	0.04	0.97	0.98	-0.01
FPC	CH40	-0.06(-0.21-0.10)	-0.29(-0.75—0.11)	-3.84	**< 0.01**	**< 0.01**	0.52
FPC	CH41	-0.03(-0.31±0.20)	-0.09(-0.21±0.17)	-0.25	0.80	0.96	0.03
FPC	CH42	0.09(-0.23±0.32)	-0.05(-0.28±0.10)	-1.81	0.07	0.91	0.25
FPC	CH43	-0.01(-0.22±0.34)	0.05(-0.19±0.18)	0.36	0.72	0.96	-0.05
FPC	CH44	-0.16(-0.38±0.20)	-0.06(-0.34±0.18)	0.57	0.57	0.91	-0.08
OFC	CH51	-0.04±0.27	-0.07±0.21	1.09	0.28	0.91	0.25
OFC	CH52	-0.03±0.27	-0.13±0.24	1.69	0.10	0.91	0.39
OFC	CH53	-0.04(-0.16-0.08)	-0.05(-0.16-0.04)	-0.29	0.78	0.96	0.04
OFC	CH54	-0.00(-0.13-0.23)	0.04(-0.18-0.20)	-0.18	0.86	0.97	0.02

Note：HbO is more sensitive and dependable than HbR, so only the results of HbR were discussed in this study.

Scheme 6. Deoxygenated hemoglobin concentration changes in the HC and SD performing 2-back.

Brain regions	Channel	SD group	HC group	t/Z	*P*-value	FDR	Effect Size
DLPFC	CH1	0.01(-0.08±0.13)	-0.03(-0.09±0.02)	-1.80	0.07	0.43	0.25
DLPFC	CH2	0.03(-0.08±0.13)	-0.01(-0.06±0.12)	-0.20	0.84	0.95	0.03
DLPFC	CH3	0.04(-0.07±0.20)	-0.07(-0.24±0.01)	-3.30	**<0.01**	**0.04**	0.45
DLPFC	CH4	-0.02(-0.09±0.08)	-0.02(-0.09±0.06)	-0.68	0.50	0.80	0.09
DLPFC	CH5	0.02±0.42	-0.07±0.26	1.39	0.17	0.61	0.32
DLPFC	CH7	-0.05(-0.39±0.37)	-0.06(-0.31±0.19)	0.16	0.88	0.95	-0.02
DLPFC	CH8	-0.13(-0.23±0.05)	0.05(-0.12±0.14)	2.87	0.004	0.09	-0.39
DLPFC	CH9	-0.04±0.21	-0.04±0.17	-0.17	0.86	0.95	−0.04
DLPFC	CH10	-0.17(-0.27±-0.04)	-0.13(-0.32±0.00)	0.20	0.84	0.95	-0.03
DLPFC	CH11	-0.01(-0.08±0.13)	0.01(-0.07±0.13)	0.68	0.50	0.80	-0.09
DLPFC	CH12	0.00±0.22	-0.04±0.11	0.56	0.58	0.82	0.13
DLPFC	CH13	-0.01(-0.06±0.16)	-0.05(-0.14±0.05)	-1.93	0.05	0.36	0.26
DLPFC	CH14	0.03±0.19	-0.09±0.18	2.89	0.01	0.14	0.67
DLPFC	CH19	-0.12(-0.22±0.24)	-0.09(-0.19±0.05)	0.05	0.96	0.97	-0.01
DLPFC	CH23	0.13±0.41	0.05±0.38	1.14	0.26	0.61	0.26
DLPFC	CH29	-0.13(-0.26±-0.06)	-0.09(-0.22±0.04)	1.28	0.20	0.61	-0.17
DLPFC	CH34	-0.12±0.24	-0.12±0.21	0.04	0.97	0.97	0.01
DLPFC	CH50	-0.12(-0.35±0.11)	-0.07(-0.20±0.01)	-0.07	0.94	0.97	0.01
DLPFC	CH55	-0.07(-0.35±0.18)	0.06(-0.31±0.30)	1.04	0.30	0.61	-0.14
VLPFC	CH17	-0.08±0.27	-0.11±0.17	0.55	0.59	0.82	0.13
VLPFC	CH18	0.00(-0.15±0.23)	-0.14(-0.29±0.08)	-2.12	0.03	0.32	0.29
VLPFC	CH24	-0.12(-0.38±0.08)	-0.21(-0.37±0.11)	-0.21	0.83	0.95	0.03

VLPFC	CH25	-0.12(-0.43-0.23)	-0.06(-0.34-0.25)	0.58	0.56	0.82	−0.08
VLPFC	CH28	0.00±0.22	-0.10±0.15	2.11	0.04	0.34	0.49
VLPFC	CH35	0.01(-0.16±0.21)	-0.03(-0.17±0.09)	-1.29	0.20	0.61	0.18
VLPFC	CH39	-0.13(-0.46±0.18)	-0.03(-0.26±0.27)	1.29	0.20	0.61	-0.18
VLPFC	CH45	0.07±0.49	0.13±0.50	-0.66	0.51	0.80	−0.15
FPC	CH20	-0.02(-0.29±0.32)	-0.10(-0.27±0.19)	-0.34	0.73	0.95	0.05
FPC	CH21	-0.07±0.40	-0.14±0.30	1.22	0.23	0.61	0.28
FPC	CH22	0.01±0.47	-0.13±0.46	1.60	0.11	0.53	0.37
FPC	CH30	-0.01±0.23	-0.04±0.27	0.80	0.43	0.80	0.19
FPC	CH31	-0.05(-0.14±0.06)	-0.09(-0.20±0.07)	-0.64	0.52	0.80	0.09
FPC	CH32	0.02±0.21	-0.05±0.17	1.10	0.28	0.61	0.26
FPC	CH33	-0.04±0.23	-0.07±0.23	0.76	0.45	0.80	0.18
FPC	CH40	-0.16(-0.37±0.03)	-0.26(-1.13±-0.06)	-1.67	0.10	0.53	0.23
FPC	CH41	-0.24(-0.50-0.12)	-0.09(-0.33-0.11)	1.20	0.23	0.61	−0.16
FPC	CH42	-0.11±0.47	-0.12±0.52	0.32	0.75	0.95	0.08
FPC	CH43	-0.03(-0.27-0.29)	0.07(-0.14-0.35)	0.97	0.33	0.65	−0.13
FPC	CH44	-0.02(-0.29±0.23)	0.03(-0.26±0.18)	0.21	0.83	0.95	-0.03
OFC	CH51	-0.10±0.24	-0.12±0.25	1.09	0.28	0.61	0.25
OFC	CH52	-0.13(-0.27±0.00)	-0.19(-0.43±0.02)	-1.41	0.16	0.61	0.19
OFC	CH53	-0.05(-0.23±0.05)	-0.06(-0.26±0.02)	-0.44	0.66	0.89	0.06
OFC	CH54	-0.03(-0.17±0.16)	0.12(-0.22±0.31)	1.07	0.29	0.61	-0.14

Note：HbO is more sensitive and dependable than HbR, so only the results of HbR were discussed in this study.

Scheme 7. Total oxygenated hemoglobin concentration changes in the HC and SD performing 0-back.

Brain regions	Channel	SD group	HC group	t/Z	*P*-value	FDR	Effect Size
DLPFC	CH1	-0.15±0.46	-0.09±0.32	-0.63	0.53	0.85	-0.15
DLPFC	CH2	-0.12(-0.28~0.11)	0.01(-0.21~0.42)	1.47	0.14	0.59	-0.20
DLPFC	CH3	-0.07(-0.38~0.20)	-0.15(-0.39~0.08)	-1.12	0.26	0.67	0.15
DLPFC	CH4	-0.18±0.41	-0.03±0.36	-1.72	0.09	0.58	-0.40
DLPFC	CH5	0.15±0.66	-0.12±0.69	1.75	0.08	0.58	0.41
DLPFC	CH7	0.48±0.90	0.46±0.95	0.12	0.91	0.92	0.03
DLPFC	CH8	-0.22(-0.66~0.14)	0.17(-0.12~0.59)	3.72	**＜0.01**	**0.01**	-0.50
DLPFC	CH9	-0.27±0.49	-0.07±0.49	-1.76	0.08	0.58	-0.41
DLPFC	CH10	-0.23±0.54	-0.08±0.60	-1.15	0.25	0.67	-0.27
DLPFC	CH11	-0.11(-0.34~0.15)	-0.06(-0.19~0.35)	1.61	0.11	0.58	-0.22
DLPFC	CH12	-0.10(-0.33~0.12)	-0.04(-0.33~0.15)	0.33	0.74	0.89	-0.05
DLPFC	CH13	-0.08(-0.36~0.11)	-0.17(-0.38~0.02)	-0.47	0.64	0.87	0.06
DLPFC	CH14	0.00(-0.30~0.20)	-0.14(-0.35~0.08)	-1.10	0.27	0.67	0.15
DLPFC	CH19	-0.11(-0.52~0.56)	-0.08(-0.23~0.27)	-0.11	0.91	0.92	0.02
DLPFC	CH23	0.67(0.05~1.14)	0.40(0.00~1.14)	-0.55	0.59	0.85	0.07
DLPFC	CH29	-0.14±0.56	-0.02±0.61	-0.92	0.36	0.72	-0.21
DLPFC	CH34	-0.03(-0.37~0.17)	-0.07(-0.26~0.11)	0.19	0.85	0.92	-0.03
DLPFC	CH50	-0.28(-0.60~0.42)	-0.11(-0.73~0.21)	0.10	0.92	0.92	-0.01
DLPFC	CH55	0.83±1.15	0.64±1.41	0.65	0.52	0.85	0.15
VLPFC	CH17	-0.21±0.61	-0.08±0.37	-1.10	0.28	0.67	-0.26
VLPFC	CH18	-0.01(-0.24~0.39)	0.01(-0.39~0.43)	-0.33	0.74	0.89	0.05
VLPFC	CH24	0.20±1.00	0.47±0.67	-1.39	0.17	0.65	-0.32

VLPFC	CH25	0.87(0.36~1.47)	0.67(0.24~1.40)	-0.59	0.56	0.85	0.08
VLPFC	CH28	-0.05±0.53	-0.17±0.44	1.02	0.31	0.67	0.24
VLPFC	CH35	0.07(-0.25~0.35)	0.14(-0.30~0.44)	-0.33	0.74	0.89	0.05
VLPFC	CH39	1.14±1.12	1.03±1.17	0.41	0.68	0.89	0.10
VLPFC	CH45	0.71(0.14~1.20)	0.39(-0.20~0.91)	-1.58	0.11	0.58	0.21
FPC	CH21	0.18(-0.23~0.53)	-0.10(-0.42~0.25)	-1.78	0.08	0.58	0.24
FPC	CH22	0.73±1.01	0.34±0.99	1.68	0.10	0.58	0.39
FPC	CH30	-0.01±0.47	-0.02±0.48	0.11	0.92	0.92	0.03
FPC	CH31	0.07±0.56	-0.02±0.66	0.57	0.57	0.85	0.13
FPC	CH32	0.03(-0.14~0.26)	-0.07(-0.24~0.17)	-1.03	0.30	0.67	0.14
FPC	CH33	-0.08(-0.30~0.07)	0.00(-0.22~0.15)	1.15	0.25	0.67	-0.16
FPC	CH40	0.09(-0.20~0.47)	-0.13(-0.73~0.35)	-1.53	0.13	0.59	0.21
FPC	CH41	0.43±1.16	0.30±0.91	0.52	0.61	0.85	0.12
FPC	CH42	0.53±0.95	0.40±0.86	0.64	0.53	0.85	0.15
FPC	CH43	0.78±0.98	0.61±1.18	0.66	0.51	0.85	0.15
FPC	CH44	0.27(-0.20~0.97)	0.17(-0.42~1.10)	-0.99	0.32	0.67	0.13
OFC	CH51	-0.13(-0.56~0.40)	-0.13(-0.60~0.24)	-0.17	0.87	0.92	0.02
OFC	CH52	0.05±0.59	-0.16±0.88	1.22	0.23	0.67	0.28
OFC	CH53	0.10(-0.19~0.40)	-0.01(-0.42~0.33)	-0.68	0.50	0.85	0.09
OFC	CH54	0.73±0.88	0.76±1.07	-0.11	0.92	0.92	-0.03

Note ：HbO is more sensitive and dependable than HbR， so only the results of HbR were discussed in this study

Scheme 8. Total oxygenated hemoglobin concentration changes in the HC and SD performing 1-back.

Brain regions	Channel	SD group	HC group	t/Z	*P*-value	FDR	Effect Size

DLPFC	CH1	-0.10(-0.37~0.09)	-0.10(-0.28~0.12)	0.43	0.67	0.92	-0.06
DLPFC	CH2	-0.10(-0.27~0.12)	-0.01(-0.21~0.42)	1.35	0.18	0.64	-0.18
DLPFC	CH3	-0.03(-0.36~0.20)	-0.14(-0.40~0.08)	-1.13	0.26	0.64	0.15
DLPFC	CH4	-0.18±0.42	-0.02±0.36	-1.73	0.09	0.52	-0.40
DLPFC	CH5	0.17±0.67	-0.10±0.68	1.78	0.08	0.52	0.41
DLPFC	CH7	0.47±0.92	0.46±0.95	0.06	0.96	0.96	0.01
DLPFC	CH8	-0.24(-0.67~0.15)	0.21(-0.14~0.60)	3.79	**< 0.01**	**0.01**	-0.51
DLPFC	CH9	-0.26±0.49	-0.06±0.49	-1.78	0.08	0.52	-0.41
DLPFC	CH10	-0.23±0.54	-0.07±0.61	-1.18	0.24	0.64	-0.28
DLPFC	CH11	-0.12(-0.34~0.14)	-0.04(-0.18~0.36)	1.60	0.11	0.52	-0.24
DLPFC	CH12	-0.10(-0.32~0.15)	-0.05(-0.33~0.16)	0.37	0.71	0.92	-0.05
DLPFC	CH13	-0.08(-0.35~0.14)	-0.18(-0.39~0.03)	-0.37	0.71	0.92	0.05
DLPFC	CH14	0.00(-0.28~0.20)	-0.14(-0.35~0.09)	-1.18	0.24	0.64	0.16
DLPFC	CH19	-0.12(-0.51~0.57)	-0.09(-0.24~0.29)	-0.08	0.94	0.96	0.011
DLPFC	CH23	0.78±1.26	0.49±0.90	1.14	0.26	0.64	0.26
DLPFC	CH29	-0.14±0.56	-0.01±0.62	-0.95	0.35	0.69	-0.22
DLPFC	CH34	-0.02(-0.37~0.16)	-0.06(-0.25~0.11)	0.25	0.80	0.93	-0.03
DLPFC	CH50	-0.27(-0.60~0.43)	-0.13(-0.75~0.23)	0.06	0.95	0.96	-0.01
DLPFC	CH55	0.82±1.16	0.65±1.42	0.58	0.57	0.91	0.13
VLPFC	CH17	-0.11(-0.48~0.12)	-0.03(-0.22~0.18)	0.81	0.42	0.80	-0.11
VLPFC	CH18	-0.01(-0.23~0.42)	0.03(-0.39~0.44)	-0.28	0.78	0.93	0.04
VLPFC	CH24	0.07(-0.39~0.64)	0.56(-0.09~0.79)	1.77	0.08	0.52	-0.24
VLPFC	CH25	1.02±1.06	0.92±1.29	0.37	0.71	0.92	0.09
VLPFC	CH28	-0.05±0.53	-0.17±0.44	1.03	0.31	0.68	0.24

VLPFC	CH35	0.06(-0.23~0.38)	0.13(-0.30~0.43)	-0.35	0.73	0.92	0.05
VLPFC	CH39	1.12±1.13	1.04±1.16	0.33	0.74	0.92	0.08
VLPFC	CH45	0.76(0.13~1.24)	0.41(-0.18~0.93)	-1.54	0.12	0.52	0.21
FPC	CH21	0.16(-0.24~0.47)	-0.10(-0.42~0.27)	-1.74	0.08	0.52	0.23
FPC	CH22	0.73±1.01	0.35±0.99	1.62	0.11	0.52	0.38
FPC	CH30	0.00±0.46	-0.02±0.49	0.12	0.91	0.96	0.03
FPC	CH31	0.07±0.56	-0.01±0.66	0.54	0.59	0.92	0.13
FPC	CH32	0.02(-0.14~0.26)	-0.05(-0.24~0.16)	-1.02	0.31	0.68	0.14
FPC	CH33	-0.08(-0.31~0.07)	0.01(-0.21~0.15)	1.14	0.25	0.64	-0.15
FPC	CH40	0.12(-0.19~0.50)	-0.13(-0.77~0.37)	-1.57	0.12	0.52	0.21
FPC	CH41	0.42±1.16	0.32±0.91	0.42	0.68	0.92	0.01
FPC	CH42	0.54±0.97	0.41±0.86	0.61	0.54	0.91	0.14
FPC	CH43	0.78±1.00	0.62±1.17	0.64	0.52	0.91	0.15
FPC	CH44	0.31(-0.22~0.97)	0.18(-0.42~1.14)	-0.99	0.32	0.68	0.13
OFC	CH51	-0.12(-0.54~0.40)	-0.14(-0.60~0.22)	-0.23	0.82	0.93	0.03
OFC	CH52	0.05±0.60	-0.17±0.90	1.24	0.22	0.64	0.29
OFC	CH53	0.09(-0.23~0.41)	-0.02(-0.42~0.33)	-0.73	0.47	0.85	0.10
OFC	CH54	0.73±0.89	0.76±1.07	-0.15	0.88	0.96	-0.04

Note：HbO is more sensitive and dependable than HbR，so only the results of HbR were discussed in this study

Scheme 9. Total oxygenated hemoglobin concentration changes in the HC and SD performing 2-back.

Brain regions	Channel	SD group	HC group	t/Z	*P*-value	FDR	Effect Size
DLPFC	CH1	-0.10(-0.38~0.10)	-0.10(-0.26~0.13)	0.40	0.69	0.92	-0.05
DLPFC	CH2	-0.08(-0.25~0.14)	-0.03(-0.21~0.42)	1.30	0.19	0.69	-0.18

DLPFC	CH3	-0.03(-0.34~0.21)	-0.16(-0.41~0.09)	-1.20	0.23	0.69	0.16
DLPFC	CH4	-0.18±0.43	-0.02±0.36	-1.69	0.10	0.67	-0.39
DLPFC	CH5	0.19±0.69	-0.09±0.68	1.76	0.08	0.67	0.41
DLPFC	CH7	0.46±0.93	0.46±0.94	-0.01	0.99	0.99	-0.00
**DLPFC**	**CH8**	**-0.23(-0.65~0.17)**	**0.24(-0.13~0.62)**	**3.74**	**＜0.01**	**0.01**	-0.51
DLPFC	CH9	-0.25±0.49	-0.05±0.49	-1.76	0.08	0.67	-0.41
DLPFC	CH10	-0.22±0.54	-0.06±0.61	-1.20	0.24	0.69	-0.28
DLPFC	CH11	-0.12(-0.33~0.15)	-0.03(-0.18~0.36)	1.61	0.11	0.67	-0.22
DLPFC	CH12	-0.09(-0.32~0.18)	-0.06(-0.33~0.17)	0.31	0.76	0.92	-0.04
DLPFC	CH13	-0.08(-0.34~0.15)	-0.19(-0.41~0.03)	-0.41	0.69	0.92	0.06
DLPFC	CH14	0.00(-0.27~0.21)	-0.14(-0.35~0.09)	-1.20	0.23	0.69	0.16
DLPFC	CH19	-0.11(-0.51~0.59)	-0.08(-0.25~0.29)	-0.11	0.91	0.96	0.02
DLPFC	CH23	0.62(0.07~1.19)	0.42(0.04~1.17)	-0.47	0.64	0.92	0.06
DLPFC	CH29	-0.13±0.56	0.00±0.63	-0.94	0.35	0.74	-0.22
DLPFC	CH34	0.00(-0.36~0.15)	-0.04(-0.24~0.11)	0.28	0.78	0.92	-0.17
DLPFC	CH50	-0.25(-0.61~0.46)	-0.13(-0.76~0.23)	0.05	0.96	0.98	-0.01
DLPFC	CH55	0.81±1.16	0.65±1.41	0.53	0.60	0.92	0.12
VLPFC	CH17	-0.10(-0.47~0.13)	-0.03(-0.23~0.20)	0.74	0.46	0.91	-0.1
VLPFC	CH18	0.01(-0.23~0.46)	0.05(-0.40~0.46)	-0.29	0.78	0.92	0.04
VLPFC	CH24	0.19±1.03	0.48±0.68	-1.47	0.15	0.68	-0.34
VLPFC	CH25	0.81(0.44~1.44)	0.71(0.23~1.34)	-0.57	0.57	0.92	0.08
VLPFC	CH28	-0.04±0.53	-0.16±0.45	1.06	0.29	0.69	0.25
VLPFC	CH35	0.06(-0.22~0.43)	0.12(-0.31~0.45)	-0.40	0.69	0.92	0.05
VLPFC	CH39	1.10±1.13	1.03±1.16	0.25	0.80	0.92	0.06

VLPFC	CH45	0.78±1.10	0.50±1.20	1.05	0.30	0.69	0.25
FPC	CH21	0.17(-0.21~0.46)	-0.08(-0.44~0.29)	-1.55	0.12	0.67	0.21
FPC	CH22	0.72±1.01	0.36±1.00	1.54	0.13	0.67	0.36
FPC	CH30	0.01±0.46	-0.01±0.49	0.14	0.89	0.96	0.03
FPC	CH31	0.07±0.57	0.00±0.66	0.51	0.61	0.92	0.12
FPC	CH32	0.03(-0.13~0.26)	-0.02(-0.24~0.15)	-1.07	0.29	0.69	0.14
FPC	CH33	-0.07(-0.30~0.08)	0.01(-0.21~0.16)	1.05	0.29	0.69	-0.14
FPC	CH40	0.13(-0.19~0.52)	-0.12(-0.79~0.37)	-1.54	0.12	0.67	0.21
FPC	CH41	0.41±1.15	0.32±0.92	0.35	0.73	0.92	0.08
FPC	CH42	0.53±0.97	0.41±0.87	0.58	0.56	0.92	0.14
FPC	CH43	0.77±1.00	0.63±1.15	0.59	0.56	0.92	0.14
FPC	CH44	0.32(-0.22~0.96)	0.16(-0.41~1.18)	-0.95	0.34	0.74	0.13
OFC	CH51	-0.11(-0.53~0.41)	-0.10(-0.61~0.21)	-0.24	0.81	0.92	0.03
OFC	CH52	0.06±0.60	-0.17±0.91	1.27	0.21	0.69	0.30
OFC	CH53	0.07(-0.26~0.45)	-0.01(-0.42~0.33)	-0.71	0.48	0.91	0.10
OFC	CH54	0.73±0.90	0.77±1.07	-0.20	0.84	0.93	-0.05

Note：HbO is more sensitive and dependable than HbR，so only the results of HbR were discussed in this study
